# Prevalence and causes of vision impairment and blindness in the Russian ural eye and medical study

**DOI:** 10.1038/s41598-020-69439-4

**Published:** 2020-07-24

**Authors:** Mukharram M. Bikbov, Gyulli M. Kazakbaeva, Rinat M. Zainullin, Timur R. Gilmanshin, Ildar F. Nuriev, Artur F. Zaynetdinov, Dilya F. Yakupova, Yulia V. Uzianbaeva, Songhomitra Panda-Jonas, Svetlana R. Mukhamadieva, Renat I. Khikmatullin, Said K. Aminev, Inga I. Arslangareeva, Jost B. Jonas

**Affiliations:** 1 Ufa Eye Research Institute, 90 Pushkin Street, Ufa, Bashkortostan Russia 450077; 2 Department of Ophthalmology, Medical Faculty Mannheim of the Ruprecht-Karls-University of Heidelberg, Theodor-Kutzerufer 1, 68167 Mannheim, Germany

**Keywords:** Public health, Eye diseases

## Abstract

To assess prevalence of mild vision impairment (MVI; best corrected visual acuity (BCVA) < 6/12 to 6/18 in the better eye), moderate-to-severe vision impairment (MSVI; BCVA < 6/18 but ≥ 3/60) and blindness (BCVA < 3/60) in a local population in Russia, we conducted the population-based Ural Eye and Medical Study. Out of 7,328 eligible individuals aged 40 + years, 5,899 (80.5%) individuals participated. MVI was present in 184 (3.1%; 95% confidence interval (CI) 2.7, 3.6) individuals, MSVI in 182 (3.1%; 95% CI 2.7, 3.5) individuals, and 11 individuals (0.19%; 95% CI 0.008, 0.30) were blind. Causes for MSVI were cataract (n = 109; 59.9%), late stage of age-related macular degeneration (n = 14; 7.7%; geographic atrophy and neovascular AMD in 7 (3.8%) individuals) each), myopic maculopathy (n = 11; 6.0%), glaucoma (n = 9; 4.9%), non-glaucomatous optic nerve damage (n = 5; 2.7%), and diabetic retinopathy (n = 4; 2.2%). Causes for blindness were cataract (n = 3; 27.3%), myopic maculopathy (n = 2; 18.2%), retinal dystrophies (n = 2; 18.2%), glaucoma (n = 1; 9.1%), and corneal scars (n = 1; 9.1%). Higher prevalence of MSVI/blindness was associated with age (*P* < 0.001; odds ratio (OR)1.10; 95% CI 1.08, 1.12), male gender (*P* < 0.001; OR 2.32; 95% CI 1.47, 3.66), educational level (*P* < 0.001; OR 0.83; 95% CI 0.76,0.92), manual grip force (*P* < 0.001; OR 0.94; 95% CI 0.92, 0.96), diabetes prevalence (*P* = 0.006; OR 1.67; 95% CI 1.08, 2.56) and axial length (*P* < 0.001; OR 1.43; 95% CI 1.26,1.62). In this population from Bashkortostan/Russia, prevalence of MVI, MSVI and blindness was 3.1%, 3.1% and 0.19%, respectively. Cataract was the most frequent cause of reversible vision impairment, while AMD, myopic maculopathy and glaucoma were the most common reasons for irreversible vision impairment.

## Introduction

Vision impairment and blindness are of utmost importance for the quality of life for the individual and for public health in general^1,2^. Recent global and regional meta-analyses summarizing available data on visual impairment and blindness have revealed that out of 7.33 billion people living in 2015, approximately 36.0 million (80% Uncertainty interval (UI): 12.9–65.4 million; 55% female) individuals globally were blind, 216.6 million (80% UI: 98.5–359.1 million) individuals were moderately to severely vision impaired, and 188.5 million individuals (80% UI: 64.5–350.2 million; 54% female) were mildly vison impaired^3,4^. These meta-analyses also showed that for Russia comprehensive data on vision impairment and blindness have only scarcely been available so far, although Russia is by area the largest, and by population one of the largest, countries worldwide^5,6^. In addition, most of the previous population-based studies on VI and blindness have only rudimentarily assessed associations between the prevalence of VI and blindness and other ocular and systemic parameters^3,4,6^. We therefore conducted the Ural Eye and Medical Study (UEMS) to examine the prevalence of VI and blindness in a local population in Bashkortostan/Russia and to explore associations between VI and blindness and other systemic parameters. The UEMS is a population-based study conducted with the aim to explore the prevalence and associated factors of major eye disorders and general medical diseases such as arterial hypertension, diabetes mellitus, chronic kidney disease and chronic obstructive pulmonary disease^7–9^.

## Methods

The UEMS is a population-based investigation which was performed in the Russian republic of Bashkortostan at the southwestern end of the Ural Mountains in the study period from 2015 to 2017^7–9^. Study regions were Ufa as capital of Bashkortostan and a rural region in the Karmaskalinsky District in a distance of 65 km from Ufa. Ufa is geographically located at 54° 44′ North and 55° 58′ East about 1,400 km East of Moscow. With a population was 1.06 million inhabitants, making it the eleventh most populous city in Russia, Ufa is the industrial, economic, scientific and cultural center of Bashkortostan. The republic of Bashkortostan is located between the Volga River and the Ural Mountains, North East of the Caspian See and North West of Kazakhstan. With a population of 4 million people, Bashkortostan is the most populous republic in Russia. Inclusion criteria for the study were living in the study regions and an age of 40 years or older. The Ethics Committee of the Academic Council of the Ufa Eye Research Institute approved the study design and confirmed that the study adhered to the Declaration of Helsinki, and all participants gave an informed written consent. The study size was based on calculations taking into account a prevalence of 1% to 10% of the major diseases to be addressed, and a response rate of about 80%.

The study participants were brought with a bus from the villages or from their urban habitations to the institute. All examinations were conducted in the Ufa Eye Research Institute in Ufa by a team of about 20 trained and surveyed technicians and ophthalmologists. All study participants underwent a series of examinations, which started with a detailed interview performed by trained social workers. The interview included more than 250 standardized questions on the socioeconomic background, smoking habits and alcohol consumption, physical activity, diet, depression and anxiety, and known diagnosis and therapy of major diseases. For the assessment of depression and anxiety, we used Zung’s Self-Rating Depression Scale and the State-Trait Anxiety Inventory (STAI)^10^. The examinations further included anthropometry, blood pressure measurement, handgrip dynamometry, spirometry, and biochemical analysis of blood samples taken under fasting conditions. We applied the Guidelines for Accurate and Transparent Health Estimates Reporting (GATHER statement guidelines) for collecting the data^11^. According to the new guidelines of the American Heart Association, we differentiated between normal blood pressure, elevated blood pressure, stage 1 and stage 2 of arterial hypertension, and a hypertensive crisis^12^. Diagnostic criteria for diabetes mellitus were a fasting serum glucose concentration of ≥ 7.0 mmol/L or a self-reported history of physician-based diagnosis or therapy of diabetes mellitus. We described the study design in detail recently^7–9^.

The ophthalmologic examinations started with the measurement of visual acuity, performed by ophthalmologists or optometrists. Under standardized conditions and using modified Early Treatment of Diabetic Retinopathy Study (ETDRS) charts (Light House Low Vision Products, New York, NY) at a distance of 4 m, we determined first uncorrected visual acuity without any glasses. After performing automated refractometry (Auto-2Ref/Keratometer HRK-7000A HUVITZ Co, Ltd., Gyeonggi-do, Korea) and subjective refractometry, we measured the best corrected visual acuity (BCVA). If the optotypes could not be read at a distance of 4 m, we continued with optotypes held at a distance of 1 m. If the optotypes could not be read at that distance, we tested the ability of finger counting and the detectability of hand movements at a distance of 1 m or 50 cm. If hand movements could not be seen, we assessed the light perception with correct or incorrect projection. We could not reliably measure presenting visual acuity with the glasses the patients usually worn, since many study participants had not brought their glasses, in particular those for reading, to the examination.

Using the World Health Organization criteria, we defined mild vision impairment (MVI) as BCVA of < 6/12 to 6/18 inclusive in the better eye, moderate to severe vision impairment (MSVI) as BCVA of < 6/18 but ≥ 3/60 in the better eye, and blindness as BCVA of < 3/60 in the better eye.

After measuring visual acuity, we performed a perimetric examination of the visual field (PTS 1000 Perimeter, Optopol Technology Co., Zawercie, Poland; screening test program with 82 test points and an extension of 50° in all directions). Using a Scheimflug camera (Pentacam HR, Typ70900, OCULUS, Optikgeräte GmbH Co., Wetzlar, Germany), we carried out an anterior chamber morphometry for measurement of the biometric parameters of the cornea and anterior chamber. A slit lamp-based biomicroscopy of the anterior ocular segment followed. All these examinations were carried out by a fellowship-trained ophthalmologist. We measured the intraocular pressure by non-contact tonometry (Tonometer Kowa KT-800, Kowa Company Ltd., Hamamatsu City, Japan), and repeated the measurement, if the readings were higher than 21 mmHg. After inducing medical mydriasis (tropicamide 0.8% and phenylephrine 5% given twice in a 10-min interval), a second slit lamp examination was performed to assess the presence of pseudoexfoliation of the lens. We paid special attention to the anterior surface of the lens and the pupillary margin to search for signs of pseudoexfoliation syndrome. Digital photographs of the cornea and lens were taken for the assessment of lens opacities (Topcon slit lamp and camera, Topcon Corp. Tokyo, Japan). We differentiated nuclear lens opacities into 6 grades using the classifying scheme for cataract of the Age-Related Eye Disease Study^13^. We defined the presence of nuclear cataract as a nuclear cataract grade of 3 or higher. Cortical lens opacities and posterior subcapsular opacities were graded using photographs taken by retro-illumination (Topcon slit lamp and camera, Topcon Corp. Tokyo, Japan). Using a grid, we measured the percentage area of opacity. The optic disc and macula were examined on digital monoscopical 60° photographs (VISUCAM 500, Carl Zeiss Meditec AG, Jena, Germany) and by spectral-domain optical coherence tomography (OCT) (RS-3000, NIDEK co., Ltd., Aichi Japan).

Using the OCT scans, we measured the peripapillary retinal nerve fiber layer thickness, the width and shape of the neuroretinal rim and the depth of the optic cup, and the thickness of the retina as a whole and stratified into various retinal layers in the foveola and in the perifoveal region. The degree of fundus tessellation was examined on the fundus photographs centered on the macula and centered on the optic nerve head^14^. Fundus tessellation was differentiated between grade “0” (no tessellation) and grade “3” (marked tessellation). AMD was defined as suggested by the recent Beckman Initiative for Macular Research Classification Committee^15^. The clinical classification system was based on fundus lesions assessed within two disc diameters of the fovea in persons. Early AMD was characterized by medium-sized drusen (diameter ≥ 63 to < 125 μm) without pigmentary abnormalities. We defined intermediate AMD by the presence of large drusen or by pigmentary abnormalities associated with at least medium-sized drusen. Late AMD showed either neovascular AMD or geographic atrophy. The presence of small drusen (diameter < 63 μm), also termed drupelets, were considered to represent normal aging changes. Glaucoma was defined by morphological criteria as described by Foster and colleagues, such as a vertical cup/disc diameter ratio or an inter-eye asymmetry in the vertical cup/disc diameter ratio of ≥ 97.5th percentile for the normal population, or a neuroretinal rim width reduced to ≤ 0.1 (10%) of the vertical optic disc diameter (between 11 and 1 o’clock or 5 and 7 o’clock)^16^. In addition, a definite visual field defect consistent with glaucoma was used for the diagnosis. The differentiation between open-angle glaucoma and primary angle-closure glaucoma was based on the appearance of the anterior chamber angle on the images taken with the Pentacam camera. Open-angle glaucoma was characterized by an open anterior chamber angle, in addition to a normal depth of the anterior chamber as assessed by slit-lamp biomicroscopy. Using the definition by Foster and associates, the anterior chamber angle was defined as occludable in the case of angle-closure glaucoma if ≥ 270 degrees of the posterior trabecular meshwork could not be seen upon gonioscopy. The equivalent on the Pentacam images was an anterior chamber angle configuration in which the peripheral iris had contact with the peripheral cornea over a circumference of ≥ 270 degrees or in which the anterior chamber angle was so narrow over a circumference of ≥ 270 degrees, that it appeared unlikely, that a gonioscopical view would have been possible to visualize the posterior trabecular meshwork.

Criterion to be included into the present study was the availability of measurements of visual acuity. Using a statistical software package (SPSS, 25.0, IBM, Chicago, IL, USA), we calculated the mean values and 95% confidence intervals (CI) of the prevalences of MVI, MSVI and blindness and assessed associations between the prevalences of MVI, MSVI or blindness with other ocular and systemic parameters, first in a binary univariate regression analysis, followed by a multivariable binary regression analysis. We included as independent parameters all those variables that were associated (*P* ≤ 0.10) with the prevalence of VI or blindness in the univariate analyses, and we expressed the associations as odds ratios (OR).

## Results

The study included 5,899 (80.5%) out of 7,328 eligible individuals (mean age: 59.0 ± 10.7 years; range 40–94 years). Out of 5,899 individuals primarily participating in the Ural Eye and Medical Study, the present investigation included 5,893 (99.9%) individuals (3,315 (56.3%) women) with assessment of visual acuity, with 3,399 participants (57.7%) living in the rural region and 2,494 (42.3%) individuals in the urban region. Their mean age was 59.0 ± 10.7 years (median: 58 years; range 40–94 years), and their axial length was 23.3 ± 1.1 mm (median: 23.2 mm; range 19.8–32.87 mm). Among the study population, 17 (0.3%) individuals were illiterate, 104 (1.8%) had passed the 5th class, 593 (10.1%) had passed the 6th class, 659 (11.2%) had passed the 10th class, 782 (13.3%) had passed the 11th class, 2048 (34.8%) were graduates, 52 (0.9%) were post graduates, and 1637 (27.8%) individuals had a specialized secondary education. The study group included 1,185 (20.1%) Russians, 1,059 (18.0%) Bashkirs, 2,437 (41.4%) Tartars, 587 (10.0%) Chuvash, 21 (0.4%) Mari and 604 (10.3%) individuals of other ethnicities or individuals without indicating their ethnicity. The study population did not vary markedly in the distribution of sex and age as compared to the data about the Russian population collected in the census from 2010^17^. Age (*P* = 0.94), axial length (*P* = 0.65) and gender (*P* = 0.70) did not vary significantly between the study participants with assessment of visual acuity and the individuals without visual acuity measurement. Within the study group the prevalence of the systemic diseases such as diabetes mellitus, arterial hypertension (stage 1 +), chronic kidney disease (estimated glomerular filtration rate ≤ 60 mL/min/1.73 m^2^) and chronic airflow obstruction (spirometrically defined by a cut-off value of the forced expiratory volume in one second (FEV1)/forced vital capacity (FVC) ratio of less than 0.7) was 687/5,893 (11.7%), 4,981/5,893 (84.6%), 1673/5,893 (28.4%), and 369/5,893 (6.3%), respectively. The prevalence of ocular diseases such as cataract, glaucoma, diabetic retinopathy and age-related macular degeneration was 2,178/5,882 (37.0%), 208/5,543 (3.5%), 101/4,898 (1.7%) and 705/5,125 (13.8%), respectively.

Among the 5,893 study participants, 184 (3.1%; 95% CI 2.7, 3.6) individuals fulfilled the definition of MVI, 182 (3.1%; 95% CI 2.7, 3.5), individuals fulfilled the definition of MSVI in the better eye or under binocular conditions, and 11 individuals (0.19%; 95% CVI 0.008, 0.030) fulfilled the definition of blindness in the better eye or under binocular conditions (Table 1).

**Table 1:** Prevalence of mild vision impairment (best corrected visual acuity (BCVA) < 6/12 to 6/18 inclusive in the better eye), moderate to severe vision impairment (BCVA < 6/18 but ≥ 3/60 in the better eye) and blindness (BCVA < 3/60) in the Ural Eye and Medical Study, stratified by age and gender.

Age group	Numbers	Mild vision impairment	Moderate to severe vision impairment	Blindness
**Men**				
40 to < 50 years	573	5 (0.9%; 95% CI 0.1, 1.6)	6 (1.1%; 95% CI 0.2, 1.9)	0
50 to < 60 years	927	17 (1.8%; 95% CI 1.0, 2.7)	10 (1.1%; 95% CI 0.4, 1.7)	2 (0.2%; 95% CI 0.0, 0.5)
60 to < 70 years	697	19 (2.7%; 95% CI 1.5, 3.9)	23 (3.3%; 95% CI 2.0, 4.6)	1 (0.1%; 95% CI 0.0, 0.4)
70 to < 80 years	302	32 (10.6%; 95% CI 7.1, 14.0)	27 (8.9%; 95% CI 5.7, 12.1)	2 (0.7%; 95% CI 0.0, 1.6)
80 + years	78	12 (15.4%; 95% CI 7.2, 23.6)	15 (19.2%; 95% CI 10.3, 28.2)	0
**Women**				
40 to < 50 years	668	1 (0.2%; 95% CI 0.0, 0.4)	5 (0.8%; 95% CI 0.1, 1.4)	0
50 to < 60 years	1,042	14 (1.3%; 95% CI 0.6, 2.0)	7 (0.7%; 95% CI 0.2, 1.2)	1 (0.1%; 95% CI 0.0, 0.3)
60 to < 70 years	1,014	27 (2.7%; 95% CI 1.7, 3.7)	26 (2.6%; 95% CI 1.6, 3.5)	1 (0.1%; 95% CI 0.0, 0.3)
70 to < 80 years	468	37 (7.9%; 95% CI 5.5, 10.4)	29 (6.2%; 95% CI 4.0, 8.4)	4 (0.9%; 95% CI 0.0, 1.7)
80 + years	123	20 (16.3%; 95% CI 9.7, 22.9)	34 (27.6%; 95% CI 19.6, 35.7)	0

**Figure 1:**
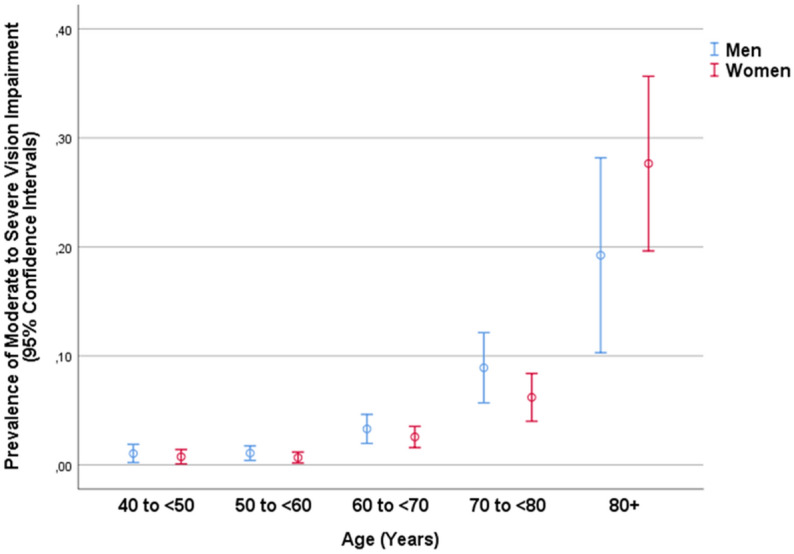
Graph showing the association between the prevalence of moderate to severe vision impairment (visual acuity < 6/18 but ≥ 3/60 in the better eye) in the Ural Eye and Medical Study, stratified by age and gender.

**Figure 2:**
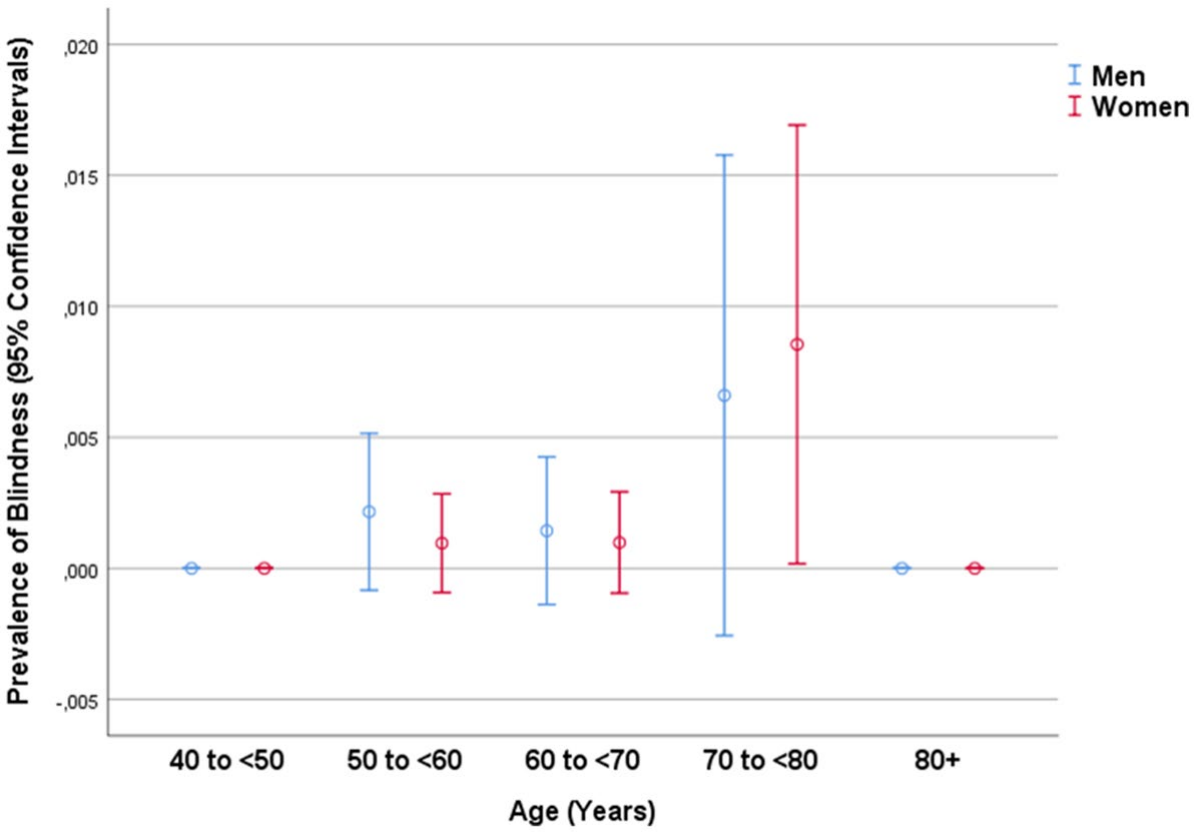
Graph showing the association between the prevalence of blindness (visual acuity < 3/60) in the Ural Eye and Medical Study, stratified by age and gender.

**Figure 3:**
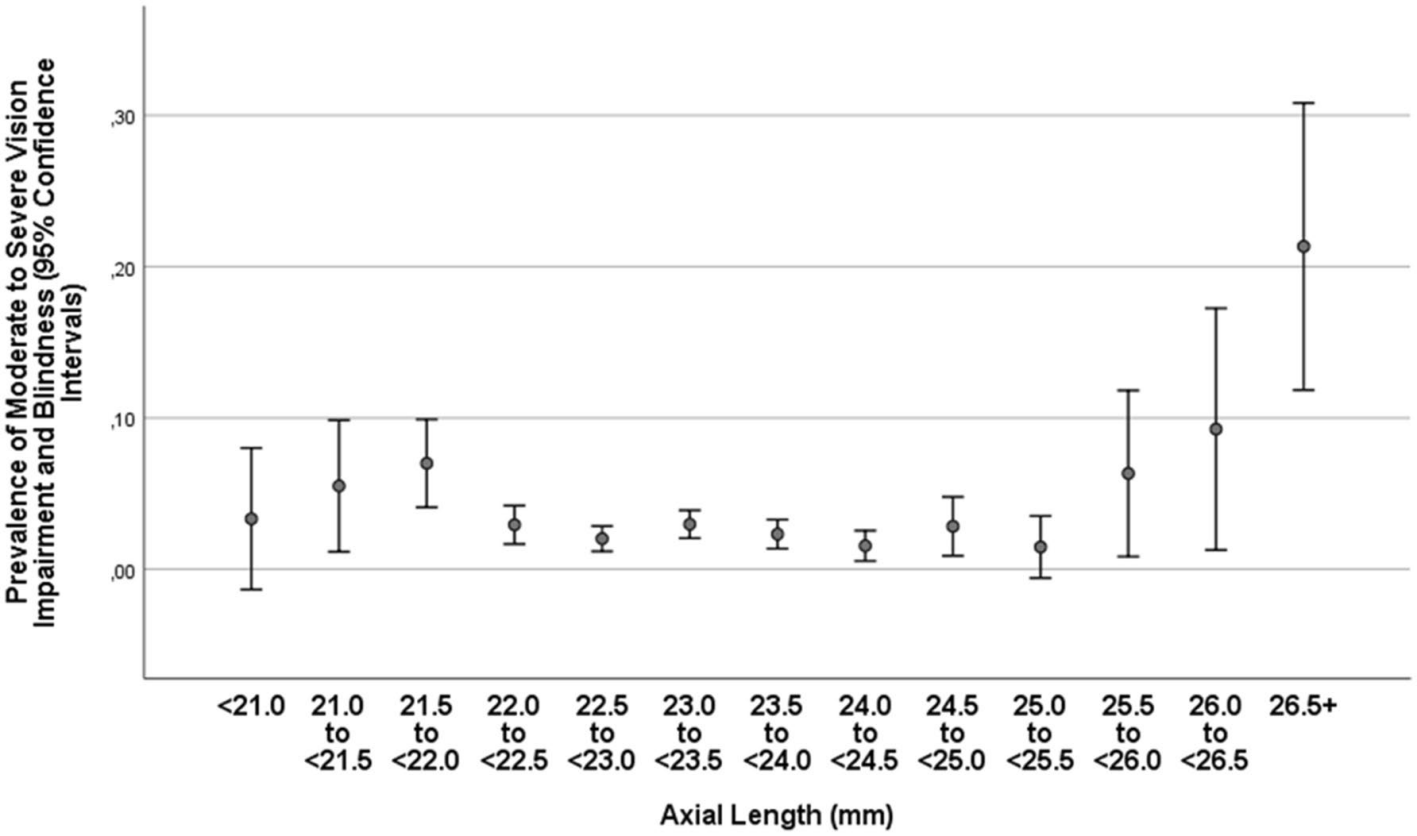
Graph showing the association between the prevalence of moderate to severe vision impairment (visual acuity < 6/18 but ≥ 3/60 in the better eye) and blindness (visual acuity < 3/60) combined, stratified by axial length, in the Ural Eye and Medical Study, stratified by age and gender.

In univariate analysis, the prevalence of MSVI and blindness combined increased significantly with the systemic parameters of older age (*P* < 0.001), urban region of habitation (*P* = 0.03), lower body height (*P* = 0.002), body weight (*P* = 0.008) and body mass index (*P* = 0.007), lower circumference of waist (*P* = 0.03) and hip (*P* < 0.001), lower level of education (*P* < 0.001), lower socioeconomic score (*P* < 0.001), lower physical activity score (*P* < 0.001), higher depression score (*P* = 0.01) and higher anxiety score (*P* = 0.01), lower intake of fruits (*P* = 0.04) and vegetables (*P* = 0.04), higher systolic blood pressure (*P* < 0.001), lower serum concentration of hemoglobin (*P* = 0.001), higher serum concentration of rheumatoid factor (*P* = 0.008), glucose (*P* < 0.001) and urea (*P* = 0.001), a higher erythrocyte sedimentation rate (*P* = 0.004), a lower erythrocyte count (*P* < 0.001), a higher prevalence of diabetes (*P* < 0.001), arterial hypertension (*P* < 0.001), chronic kidney disease (*P* < 0.001) and anemia (*P* = 0.005), higher hearing loss score (*P* < 0.001), lower prevalence of consumption of alcohol (*P* = 0.02), and a lower manual dynamometric grip force (*P* < 0.001); and with the ocular parameters of longer axial length (*P* = 0.002), more myopic refractive error (*P* < 0.001), and higher intraocular pressure (*P* < 0.001) (Figs. 1, 2, 3). It was not significantly associated with gender (*P* = 0.82), waist-hip circumference ratio (*P* = 0.28), Russian versus non-Russian ethnicity (*P* = 0.91), smoking (*P* = 0.16), diastolic (*P* = 0.40) and mean (*P* = 0.13) blood pressure, serum concentration of alanine aminotransferase (*P* = 0.61), aspartate aminotransferase (*P* = 0.47), aspartate aminotransferase-to- Alanine aminotransferase ratio (*P* = 0.65), total bilirubin (*P* = 0.97), high-density lipoproteins (*P* = 0.82), low-density lipoproteins (*P* = 0.73), cholesterol (*P* = 0.77), triglycerides (*P* = 0.08), creatinine (*P* = 0.18), blood leukocyte count (*P* = 0.46), and the prevalence of chronic obstructive pulmonary disease (*P* = 0.48). Including only eyes with an axial length of ≥ 25.5 mm into the analysis, the prevalence MSVI/blindness combined increased steeply with axial length (OR 1.77; 95% CI 1.30, 2.41; *P* < 0.001) (Fig. 3). For the latter analysis, we included only the moderately to highly myopic individuals (with a cut-off value of 25.5 mm), since the prevalence of myopic complications, mainly myopic maculopathy and myopia-associated optic neuropathy, markedly increases with longer axial length beyond an axial length of approximately 25.5 mm or 26 mm^18^.

In the multivariable analysis, we dropped, due to collinearity the parameters of serum concentration of glucose (prevalence of diabetes), creatinine (chronic kidney disease) and hemoglobin (anemia), body weight (body mass index), and refractive error (axial length). Due to a lack of statistical significance, we then dropped step by step the parameters of body height (*P* = 0.73), waist circumference (*P* = 0.79), anxiety score (*P* = 0.99), intake of fruits (*P* = 0.99) and vegetables (*P* = 0.50), serum concentration of rheumatoid factor (*P* = 0.63) and urea (*P* = 0.58), erythrocyte sedimentation rate (*P* = 0.58), body mass index (*P* = 0.55), region of habitation (*P* = 0.88), socioeconomic score (*P* = 0.19), physical activity score (*P* = 0.24), depression score (*P* = 0.22), prevalence of anemia (*P* = 0.91) and chronic kidney disease (*P* = 0.90), hearing loss score (*P* = 0.48), prevalence of arterial hypertension (*P* = 0.51), erythrocyte count (*P* = 0.34), alcohol consumption (*P* = 0.17), and intraocular pressure (*P* = 0.15). In the final model after adding gender to the list of independent parameters, a higher prevalence of MSVI and blindness combined was associated with older age (*P* < 0.001), male gender (*P* < 0.001), smaller hip circumference (*P* = 0.008), lower level of education (*P* < 0.001), lower manual dynamometric grip force (*P* < 0.001), higher prevalence of diabetes mellitus (*P* = 0.006), and longer axial length (*P* < 0.001) (Table 2). If we added the region of habitation to the model, it was not significantly associated with the prevalence of MSVI/blindness (*P* = 0.89).

**Table 2:** Associations (multivariable analysis) of the prevalence of moderate to severe vision impairment and blindness combined (best corrected visual acuity < 6/12) with systemic and ocular parameters.

Parameter	*P*-value	Odds ratio	95% confidence interval
Age (years)	< 0.1001	1.08	1.06, 1.102
Women/men	< 0.001	2.32	1.47, 3.66
Hip circumference (cm)	0.008	0.98	0.97, 0.995
Level of education	< 0.001	0.83	0.76, 0.92
Manual dynamometric grip force (dekaNewton)	< 0.001	0.94	0.92, 0.96
Diabetes mellitus prevalence	0.02	1.67	1.08, 2.56
Axial length (mm)	< 0.001	1.43	1.26, 1.62

The causes for MSVI were cataract (n = 109; 59.9%; 95% CI 52.7, 67.1); among them, mostly nuclear cataract (n = 24; 13.2%), mostly cortical cataract (n = 29; 15.9%), cortico-nuclear cataract (n = 53; 29.1%), subcapsular posterior cataract (n = 1; 0.5%), mature cataract (n = 1; 0.5%) and secondary cataract (n = 1; 0.5%), late stage of age-related macular degeneration (AMD) (n = 14; 7.7%; 95% CI 3.8, 11.6); among them, geographic atrophy in 7 (3.8%; 95% CI 1.0, 6.7) individuals), myopic maculopathy (n = 11; 6.0%; 95% CI 2.6, 9.5), glaucoma (n = 9; 4.9%; 95% CI 1.8, 8.1), among them angle-closure glaucoma in 2 individuals and open-angle glaucoma in 7 individuals; non-glaucomatous optic nerve damage (n = 5; 2.7%; 95% CI 0.4, 5.1), diabetic retinopathy (n = 4; 2.2%; 95% CI 0.1, 4.4), and others (Table 3). Causes for blindness were cataract (n = 3; 27.3%), myopic maculopathy (n = 2; 18.2%), retinal dystrophies (n = 2; 18.2%), glaucoma (n = 1; 9.1%) (angle-closure glaucoma), and corneal scars (n = 1; 9.1%) (Table 3).

**Table 3:** Causes for moderate to severe vision impairment (MSVI) (best corrected visual acuity (BCVA) < 6/18 but ≥ 3/60 in the better eye) and blindness (BCVA < 3/60) in the Ural eye and Medical Study, stratified by age and gender.

Disease	MSVI, numbers	MSVI, percentage	95% confidence interval	Blindness, numbers	Blindness, percentage
**Cataract**	109	59.9	52.7, 67.1	3	27.3
Mostly nuclear cataract	24	13.2		1	9.1
Mostly cortical cataract	29	15.9			
Cortico-nuclear cataract	53	29.1		1	9.1
Subcaspular posterior cataract	1	0.5			
Mature cataract	1	0.5		1	9.1
**Secondary cataract**	1	0.5			
**Age-related macular degeneration, late stage**	14	7.7	3.8, 11.6		
Geographic atrophy	7	3.8	1.0, 6.7		
Neovascular	7	3.8	1.0, 6.7		
**Myopic maculopathy**	11	6.0	2.6, 9.5		18.2
Stage 2	2	1.1			
Stage 3	3	1.6			
Stage 4	5	2.7		2	18.2
Exudative stage	1	0.5			
**Glaucoma**	9	4.9	1.8, 8.1	1	
Angle-closure glaucoma	2	1.1		1	18.2
Open-angle glaucoma	7	3.8			
**Non-glaucomatous optic nerve damage**	5	2.7	0.4, 5.1		
**Diabetic retinopathy**	4	2.2	0.1, 4.4		
**Nystagmus-associated amblyopia**	1	0.5			
**Retinal dystrophy other than retinitis pigmentosa**	1	0.5		2	18.2
**Macular hole**	1	0.5			
**Macular edema of unknown etiology**	1	0.5			
**Keratokonus**	1	0.5			
**Retinal detachment**	1	0.5			
**Branch retinal vein occlusion, non-ischemic**	1	0.5			
**Corneal scars**				1	9.1
**Unclear**	23	12.6		2	18.2

## Discussion

In our typical, ethnically mixed, study population from Bashkortostan/Russia, the prevalence of MVI, MSVI, blindness, as based on the measured of BCVA, was 3.1%, 3.1% and 0.19%, respectively. The most frequent causes for MSVI were cataract (59.9%), late stage of AMD (7.7%), myopic maculopathy (6.0%), glaucoma (4.9%), non-glaucomatous optic nerve damage (2.7%), diabetic retinopathy (2.2%), and others. Causes for blindness were cataract (27.3%), myopic maculopathy (18.2%), retinal dystrophies (18.2%), glaucoma (9.1%), and corneal scars (9.1%).

The prevalence of blindness as assessed in this population from Bashkortostan/Russia was higher than the figures found in the populations of high-income countries for which a recent meta-analysis revealed an age-standardized prevalence of blindness, based on presenting visual acuity, of 0.15% (95% CI 0.06, 0.26) for the year 2015^6^. In a similar manner; the prevalence of MSVI in our Russian population was higher than in the high-income countries with an MSVI prevalence (based on presenting visual acuity) of 1.27% (95% CI 0.55, 2.17)^6^. The MSVI and blindness prevalence in our study population was lower than in Central and South Asia, where the combined age-standardized prevalence of MVI, MSVI and blindness (based on presenting visual acuity) in 2015 were for men and women 11.7% and 12.3%, 16.3% and 17.7%, and 3.7% and 4.0%, respectively^19^. In a similar manner, the MSVI and blindness prevalence was lower in our study population than in populations from North Africa and the Middle East, where the age-standardized prevalence of MSVI and blindness (based on presenting visual acuity) for all ages was 4.62% (2.21–7.33%) and 0.95% (0.32–1.71%), respectively^20^. The same held true for Southeast Asia and Oceania with a prevalence of MVI, MSVI and blindness (based on presenting visual acuity) of 3.76 (1.03 to 7.24), 4.88 (1.56 to 9.19), and 0.72 (0.26 to 1.31) respectively^21^. In the interpretation of the data of the meta-analyses mentioned above, one has to consider that they included undercorrection of refractive error as cause of MSVI and blindness and that it makes out about 20% of the causes of blindness and about 50% of the causes of MSVI.

In our study as in other investigations, cataract was by far the most frequent cause for MSVI and blindness, followed by AMD, myopic maculopathy, glaucoma, non-glaucomatous optic nerve damage and diabetic retinopathy. In a recent global meta-analysis, the most frequent causes for worldwide MSVI were cataract (with 52.6 million individuals affected), followed by AMD (8.4 million individuals), glaucoma (4.0 million individuals) and diabetic retinopathy (2.6 million individuals), in addition to uncorrected refractive error with 116.3 million individuals affected worldwide^4^. The most frequent causes for worldwide blindness were cataract (12.6 million individuals), uncorrected refractive error (7.4 million individuals) affected and glaucoma (2.9 million)^4^. Stratified between the world regions, the lists of the most common causes for MSVI and blindness do not differ markedly between the world regions nor do they markedly vary with the list of causes of vision impairment in our study population, except for myopic maculopathy^6,19–21^. The latter was the second most frequent cause for irreversible MSVI and the second most common cause for blindness in our study population. It shows the importance of myopia as potentially blinding disease for Russia, and it also demonstrates a weakness of the previous meta-analyses of worldwide and regional MSVI and blindness in which myopic maculopathy had often not explicitly been separated from AMD within the group of macular diseases as cause for vision impairment.

The finding of myopic maculopathy as an important cause for MSVI and blindness in our study population agrees with observations made in population-based studies in East Asia. To cite an example, in the Beijing Eye Study performed as early as 2001 on a population aged 40 + years, myopic maculopathy was the most common cause for irreversible vision impairment and blindness^22^. Also other epidemiologic studies from East Asia, such as the Shihpai Eye Study and the Tajimi Study, reported similar findings^23,24^.

One may take into account that the association between the prevalence of MSVI/blindness and axial length was curvilinear, with a slight increase for the range of 21.0 to 22.0 mm of axial length, a flat part between an axial length of 22.0 mm and 25.5 mm, and a steep increase for an axial length longer than 25.5 mm (Fig. 3). If one considers the increase in the prevalence of axial myopia in the elderly population in the decades to come, one may anticipate a further increase in the prevalence of myopic maculopathy as cause for irreversible vision impairment and blindness^25^.

Factors associated with MSVI and blindness were older age, male gender, lower level of education, smaller hip circumference, lower manual dynamometric grip force, higher prevalence of diabetes mellitus and longer axial length. For each year increase in age, the risk of MSVI/blindness increased by 10% (95% CI 1.08, 1.12), the presence of diabetes mellitus increased the risk of MSVI/blindness by 70% (95% CI 1.16, 2.49), and each mm longer axial length increased the risk by 40% (95% CI 1.24, 1.57). Within the group of eyes with an axial length of ≥ 25.5 mm, the prevalence MSVI/blindness combined increased by 77% (95% CI 1.30, 2.41) for each mm increase in axial length (Fig. 3). These observations fit with the findings made in previous investigations in which increased age, axial myopia, diabetes mellitus and lower socioeconomic background were factors associated with vision impairment^3,4,6,19–23^. Interestingly, open-angle glaucoma was more often than angle-closure glaucoma cause for MSVI (Table 2). Also interestingly, the region of habitation (rural versus urban) was not significantly associated with the prevalence of MSVI and blindness. It may indicate that the access to the medical infrastructure did not differ markedly between the rural population and urban population, provided that the prevalence of risk factors for the development of MSVI and blindness were similar in both regions of habitation.

If we discuss the results of our study, we should take into account its limitations. First, we did not measure presenting visual acuity, so that we could not assess the prevalence of undercorrection of refractive error as cause for vision impairment and blindness. In previous studies, undercorrection of refractive error was a major cause for vision impairment^4^. We could thus not discuss the effect of providing glasses to correct the refractive error as the easiest, most cost-effective and probably safest way to improve vision in the population. Second, we assessed the cause of visual impairment mainly on photographs of the anterior and posterior segment of the eye, so that our investigation could not give information about amblyopia as cause for vision impairment. Since, however, bilateral deep amblyopia occurs only rarely and since we defined vision impairment and blindness by the visual status of the better eye, it might have been unlikely that bilateral amblyopia was a major cause for MSVI and blindness in our study population. Third, dense cataract might have covered diseases of the macula optic nerve so that we might have been underestimated the prevalence of these diseases in eyes with far advanced cataract. Fourth, the rate of participation and the representativeness of the study population and region are of high importance for an epidemiologic investigation. The participation rate of 80.5% in our study suggests that there was not a major bias in the participant inclusion. The study region showed a similar climate as many other regions in Russia with warm summers and long and cold winters. The ethnical structure of the population of our investigation was typical for regions of Southern Russia. As compared to North and Western Russian regions, it had a lower percentage of Russians on the total population. Interestingly, the prevalence of MSVI/blindness did not differ significantly between Russian versus non-Russian ethnicity in our study population. Our investigation had the strengths of being the first population-based investigation from Russia on the topic of research, the relatively large study sample size, and the inclusion of a multitude of systemic parameters.

In conclusion, in our typical, ethnically mixed population from Bashkortostan/Russia, the prevalence of MVI, MSVI and blindness was 3.1%, 3.1% and 0.19%, respectively. The most frequent causes for MSVI were cataract (59.9), late stage of AMD (7.7%), myopic maculopathy (6.0%), glaucoma (4.9%), non-glaucomatous optic nerve damage (2.7%), and diabetic retinopathy (2.2%). Causes for blindness were cataract (27.3%), myopic maculopathy (18.2%), retinal dystrophies (18.2%), glaucoma (9.1%), and corneal scars (9.1%). For each year increase in age and for each mm increase in axial length, the risk of MSVI/blindness increased by 10% (95% CI 1.08, 1.12) and 40% (95% CI 1.24, 1.57), respectively. Within the group of eyes with an axial length of ≥ 25.5 mm, the prevalence MSVI/blindness combined increased by 77% (95% CI 1.30, 2.41) for each mm increase in axial length.
